# The Foreign Journals: Anatomy and Physiology

**Published:** 1841-10

**Authors:** 


### PART THIRD.

### Selections from the British and Foreign Journals.

### I. THE FOREIGN JOURNALS.

### ANATOMY AND PHYSIOLOGY.

*On the Organic Causes of the Motion of the Heart.*
By Dr. J. Heine, of Germersheim.

This paper contains a remarkable case of occasional arrest of the heart's action, the appearances found after death, and the physiological deductions which the author believes they may justly warrant.

A man, thirty-six years old, was admitted into the Vienna hospital, complaining chiefly of fits, which he said depended on stoppage of the heart. It was soon found that at different times, to which there was no clue, the heart ceased to move for an interval of from four to six beats, its cessation of action giving rise to the most fearful sensations of anxiety, and to acute pain passing from both sides of the chest up the neck to the head, and there remaining fixed for some time after the fit. These attacks had already occurred frequently for a considerable time; they became more numerous after the patient's admission, and the pain of the neck and head becoming constant, he soon died comatose.

At the examination after death there were found considerable effusion of serous fluid into the cerebral ventricles without any traces of inflammation of the arachnoid, numerous small tubercles on the left hemisphere of the cerebellum, earthy and softened tubercles at the apices of the lungs, inclosure of the great cardiac nerve in a black bronchial gland as large as a hazel-nut, the same condition of the bronchial branches of the left vagus, and a remarkable increase of size of the cervical portion of the spinal marrow. All these however, with the exception of the tumour compressing the great cardiac nerve, having been often met with without the preexistence of any affection of the heart, the author ascribes to this tumour all the peculiar symptoms of the case, and draws from it the following among many deductions:

1. The great cardiac nerve is proximately the source of nervous influence for the action of the heart. 2. The interruption of its continuity destroys the heart's action, (at least its sensible action.) In the present case this interruption was only occasionally complete in consequence of the occasional enlargement and increased pressure of the tumour. 3. The muscle, even of the heart, is therefore not, as Haller supposed, independent of nervous influence. 4. Respiration, though it has an evident influence on the heart's action, has yet only a secondary influence, and is dependent for the exercise of this on integrity of the nervous power. 5. Since the occasional interruption of the continuity of the cardiac nerve in man destroys the action of the heart, and since with this interruption a shock is conveyed upwards, while at the same time the heart remains fixed, we must assume that the nerve acquires the power necessary for the maintenance of the heart's action from some part higher up. 6. The cardiac nerve has therefore, in these respects, the same relations as a cervical nerve. 7. Since this is not the only nerve going to the heart, and yet interruption of its continuity destroys the heart's action, there is no reason to believe in any communication of nervous fibres. 8. The pain in the neck and head experienced during the attack must be referred to a recoil of the nervous influence prevented from discharging itself at the periphery, and therefore turning backwards and producing a kind of shock. 9. The ganglionic system, so far as it is connected with the heart, and therefore probably in all other parts is not independent of the central organs, but is in immediate connexion with them, and receives its motor power from them, and especially from the spinal marrow.

*Muller's Archiv.* Heft iii. 1841.

---

*On the Stroke of the Heart.* By Dr. Kuerschner, of Marburg.

The results which the author here publishes he states to have been obtained from many years' observation and experiment. He draws particular attention to what Haller* and some others have cursorily remarked, but which has been generally disregarded, namely, that in the systole of the heart it not only has its apex elevated or advanced forwards, but that at the same time the apex is always pushed somewhat towards the right side, and the whole organ partially rotated on its axis, the left ventricle, which in diastole (when the animal is on its back) is directed backwards and nearly invisible, being turned forwards so that a considerable portion of its front wall is exposed to view. He also points to the fact, that very often on opening and removing the pericardium, when the animal lies on its back, the rocking motion of the heart cannot be observed, though it is contracting vigorously, and this he ascribes to the fixed position which the heart takes against the vertebral column, and which is such that it cannot recede as it ordinarily does in diastole; for he found that on raising it by the pericardium from the spine, the rocking antero-posterior motion, with the rising and falling back of the apex became again perfectly distinct. Led by this fact to the belief that the elevation of the apex is the consequence, not the precedent, of its depression, he was induced to undertake a course of experiments to determine why the apex of the heart sinks or moves backwards in diastole.

These experiments, which were again and again repeated, consisted of artificial injections and imitations of the currents of blood flowing into the heart, managed so as to produce the same motions as the heart naturally makes. They were performed on dead foxes. These were placed on their backs; their hearts were exposed by the removal of the pericardia, and through the apex of each a thread was fixed, and then carried over a pulley to a scale carrying weight sufficient to raise the heart to its natural distance from the vertebral column. Injections were now forced in different directions into the trunks of one or more of the great veins, so as to fill one or both of the auricles and ventricles; then, the piston of the syringe being raised, the fluid was again withdrawn from these cavities, and again impelled into them; and so on alternately, imitating as nearly as possible the flow of blood into, and its removal from, the auricles and ventricles.

However the experiments were varied, and into whatever vein or veins the fluid was impelled, the same general result was observed; the scale was raised by the apex of the heart moving backwards towards the spine, and the whole heart turned round with force upon its axis from right to left, so that the left ventricle was directed completely towards the spine, and the right ventricle only presented forwards. On the other hand, when the fluid was withdrawn, the contrary movements took place; the apex was elevated and the scale fell lower, and the heart turned round its axis from left to right so as to expose a portion of the left ventricle. Only in the experiments in which the left pulmonary veins were injected was anything different from these noticed; for when this was done, the heart turned during the injection from left to right, and in the opposite direction when the fluid was withdrawn. The results were the same in whatever position the body of the animal was placed; and even when it was placed with the apex of the heart directed downwards, it still moved towards the spine when fluid was impelled into it from the veins.

* Elementa Physiologiæ, vol. i. p. 389.

From these facts the author deduces, that the sinking or movement backwards of the apex of the heart is dependent on the streaming of the blood into the ventricles; and that the direction in which the blood flows from the venous trunks to the auricles determines the direction of the heart's rotatory motion. The venous current being directed principally from right to left, gives this motion the same general direction, which is that which is observed during diastole in all living animals.

The elevation of the apex is regarded by the author as secondary to its sinking, and as the result mainly of the elasticity of the great vessels attached to the base of the heart. These, when the apex is carried backwards by the current of venous blood, must, by their position, be stretched; and therefore when the ventricles contracting close the auriculo-ventricular orifices, and prevent the further influx of venous blood, the great vessels return from their condition of tension, and again carry the apex of the heart forwards. They could not however do this with a force sufficient to produce a perceptible impulse against the front of the chest, unless the ventricles were, as they are, coincidently hardened and rendered firm by their contraction, and in some measure forced forwards by their recoil from the blood which they are impelling backwards and upwards. The same forces of elasticity and recoil give the heart its second rotatory motion; for the blood moves through the arteries chiefly from right to left, and the force exerted by the left ventricle is greater than that of the right, so that its recoiling force predominates, and the heart moves, as it always does in systole, from left to right.

*Muller's Archiv.* Heft i. 1841, *p.* 103.

---

*Direct Experiments to determine whether Portions cut off from Leeches are reproduced.* By STEFANO GRANDONI.

BOSE, by whom Buffon's Natural History was continued, positively asserts the reproduction of divided leeches; in the Dictionnaire des Sciences Naturelles the contrary is stated. This contradiction with other circumstances induced the author to submit the question to the test of experiment. He divided 20 leeches between the 5th and 6th segments of their bodies, and placed half of them in glasses containing water with little stones at the bottom, and the other half in glasses containing a thin stratum of clay moistened with water. All possible care was taken to maintain them in health. After three weeks one of those that lived in the clay exhibited on its truncated extremity two white and somewhat convex gelatinous corpuscles, in the centre of which there was a more vividly coloured and transparent point; the rest of the section was somewhat rounded and covered by a very delicate membrane. These signs seemed to render it probable that the experiment was about to succeed; but a month after the leech died, and apparently from the disease of the truncated extremity. Three months after being divided, the sections of the other leeches were found covered by a dense rounded gelatinous mass, which after some time became a very fine and transparent investment, and made the surface of the divided part quite smooth. After about six months the leeches in the water with the pebbles all died one after the other, without any regeneration of the part that had been cut away. Their weight was neither diminished nor increased. At this time six of the remaining leeches were taken from the clay, in which they had till lately constantly lain buried, and put in water. These, as well as the three that were left in the clay, were then looked at several times every day; the condition of their trunks was from time to time examined with the aid of powerful lenses, and the number of their segments was counted, but no increase was observed. At the tenth month from the commencement of the experiment only the three leeches in the clay remained alive; these also died four months afterwards but without the reproduction of even the most minute ring. It was thus decided that divided leeches are not regenerated.

*Commentarij dell' Ateneo di Brescia,* 1837-8.
*Annali Universali di Medicina, Ottobre,* 1839.

*Microscopic Examination of Lymph.* By Professor Bischoff, of Heidelberg.

The fluid examined was taken from two large lymphatics in the neck of a dog. It was quite clear and pellucid, and after some time coagulated, but without assuming a reddish colour. It contained some yellowish glistening globules of no great size, having an average diameter of from $\frac{3}{10000}$ to $\frac{2}{10000}$ of a Paris inch, the largest being $\frac{4}{10000}$, the smallest $\frac{2}{10000}$. A nucleus and envelope could not be distinguished in them. They were not all quite round like the blood-globules; nor were they granular and nodulated. They were not altered by water, acetic acid, or ether; but in caustic potash they vanished immediately. Similar globules with the same reactions present themselves together with innumerable very small granules in the white contents of the thoracic duct and in the chyle.

*Müller's Archiv.* 1839, Jahresbericht, p. cxxxix.

---

*Microscopic Experiments on Blood, Plastic Lymph, Pus, and Milk.*
By Dr. Letellier, of Saint Leu.

The following are the conclusions at which the author arrives as the results of his experiments: 1st, It is not possible to prove by the microscope that the red globules of human blood are formed of a nucleus and pellicle; 2d, But the microscope and chemical agents prove that these globules are formed of an envelope probably fibrinous, and of a transparent full nucleus; 3d, This nucleus offers the chemical properties of albumen coagulated by an acid; 4th, The albumen is evidently formed of transparent grains, becoming opaque where they are precipitated upon each other by alcohol or acids; 5th, Plastic lymph which runs from wounds, carries all the elements of the blood but the red colour of the globules; 6th, Pus principally contains, (*a*,) a great number of globules of blood deprived of colouring matter, and rendered opaque; (*b*,) a small quantity of vesicles of very varied forms and dimensions, formed by cells of fibrine; (*c*,) the débris of fibrine. *L'Expérience. Oct.* 1, 1840.

---

*On Spontaneous Vomiting after Division of the Nervi Vagi.*
By Dr. J. Hoppe, of Berlin.

After a just criticism of the rather vague opinions of Valentin respecting the cause of this phenomenon, the author gives as the ground of his own decision regarding it, the following facts: 1st, He has never seen any animal vomit spontaneously after division of the nervi vagi, which had not the round stomach of carnivora, which, as Schulz observed, is so prone to the action. 2. He has very rarely seen it occur after the division of only one nerve. 3. The vomiting ensues immediately after the operation, and persists till the time of death with intervals of uncertain length. 4. The contents of the stomach were first vomited, and then mucus and gastric juice as soon as a sufficient quantity had accumulated. 5. After the animal had vomited once, it usually went on only retching till sufficient substance to be vomited had collected again. 6. The vomiting always took place during expiration, and ceased when that act was completed; and the straining continued longer only when the mucus was hard to get rid of. 7. The vomiting ceased when the muscles of the abdomen were divided. 8. There was nothing but straining when the stomach was empty at the time of dividing the nerves. 9. There was no vomiting when the animal breathed in any other way than is usual after division of the vagi. 10. And it ceased when this peculiar mode of breathing was altered by the faintness or collapse of the animal, or when by any circumstance expiration was performed without the jerking motion of the abdominal muscles. 11. The peculiar respiration of animals whose nervi vagi are divided consists of an infrequent, slow, long-continued, very forced and deep or rather great movement of the chest, which ends with a short jerking expiration. Dogs and cats during the inspiration open their mouths so wide that the whole pharynx is raised; and in expiration they close it till they have vomited or retched, and they then open it wide again. The expiration is extremely violent; the arch of the chest is forced downwards, and at the same time pulled back by the muscles of the abdomen, with such force that air passes through the nose with a loud noise. During such an expiration vomiting always takes place, but *never* at any other time, 12. The inspiratory action is so powerful that the air passes into the œsophagus. so as to distend it; but it never gets into the stomach.

From these facts there can, the author holds, be no doubt that the vomiting is merely the mechanical result of the violent contraction of the abdominal muscles, with which expiration is performed after the division of both nervi vagi, assisted as their influence is by the wide-opening of the mouth in inspiration, by which the pharynx is raised up high and the œsophagus becomes distended with air.

*Casper's Wochenschrift. November* 14, 1840.

---

*On the Development of Heat during the Incubation of Oviparous Reptiles.*
By M. Valenciennes.

At the sitting of the Royal Academy of Sciences, 19th of July, 1841, M. Valenciennes communicated the result of the observations he had made upon the female striped serpent in the *Jardin des Plantes.* It has not hitherto been positively determined if the animal heat of serpents is augmented during incubation. It would appear that the serpent in question, a native of the isles of the Indian Archipelago, would not require this increase of heat, the elevated temperature of the regions it inhabits heating the eggs without the necessity for maternal incubation. The facts established by M. Valenciennes have contradicted these presumptions. On the 5th of May last this serpent laid fifteen very large eggs, and immediately afterwards gathered herself into a spiral form, so as to surround the eggs in every part; and she did not cease to cover them during almost two months, never changing her position. When she was approached she put herself on the defensive, and appeared inclined to protect her eggs. She had made a meal of a living rabbit and a large portion of meat, but throughout the whole period of incubation she took no food, and on the 25th of May alone, about two glasses of water. On the 5th of July, the first serpent broke its egg, and in the space of twenty-four hours all the others appeared, making fifty-six days of uninterrupted incubation. At birth the young ones were about a foot in length, and the mother who had previously been so careful, abandoned them as soon as they were hatched, and paid them no more attention.

The cages in which the serpents are kept, and the coverings with which they are surrounded being artificially heated, it was not easy to distinguish the heat produced by incubation from that artificially developed, but notwithstanding this difficulty, M. Valenciennes is assured by twenty-four observations made with the greatest care, that while the temperature of the cage and coverings was 22° (72° Fah.), that of the body of the serpent covering the eggs was constantly 41° (106° Fah.) of the centigrade thermometer. It is not possible then to deny the unusual development of heat which accompanies incubation in reptiles of this class.

*Gazette Médicale de Paris.* 24 *Juillet,* 1841.

---

*On the extreme Ramifications of the Minute Arteries and Veins in the Coats of the Intestines.* By Dr. Gaddi, of Modena.

Dr. Gaddi has instituted some researches on the mode of termination of the arteries and origin of the veins in the coats of the intestines, by injecting pure water coloured with cinnabar for the arteries, and indigo for the veins, in children from two to five years of age unaffected by any intestinal disorder.

The arteries were filled by a general injection from one of the carotids, the current being directed towards the heart; the veins from one of the mesenteric. When the injection was successful, Dr. Gaddi has observed constantly that the trunks of the intestinal arteries were guided by the peritoneal duplicature to the external layer of the muscular coat of the intestinal tube; they turn around this tunic, and penetrate it, dividing into an infinite number of anastomotic arches, more and more delicate, which traverse the second muscular coat to arrive at the submucous cellular tissue. There they all terminate in a thick tuft of almost imperceptible arterioles, not one of which ever penetrates into the mucous membrane, or terminates by a free orifice. The veins on the contrary always arise on the free surface of the mucous membrane by three, or at most four veinules, which in most cases have a visible funnel-like orifice, and immediately after penetrating the substance of the membrane, they converge together and unite in the submucous tissue, in a vesicle from which a very fine venous trunk arises, which latter soon associates with the trunk of the artery; passes with it to the muscular layers, where it divides; reunites into trunks of larger and larger size; and lastly leaves the intestinal walls, and passes between the folds of peritoneum.

The tuft of very minute arterial ramusculi just described envelopes the venous vesicle [" in a sort of atmosphere,"] so that it is there that the artery discharges itself. The vesicle is the point where the arterial takes the character of venous blood; and it is also the point which puts the two systems in communication, as Dr. Gaddi was assured in the rare cases where the arterial injection penetrated to the venous trunks, to the vesicle, to the branches, and even into the cavity of the intestines. On removing and examining with the greatest care the mucous membrane alone, he has never seen an artery nor venous vesicle, but only the venous radicles in radii. This vascular disposition is constant throughout the whole length of the intestinal tube, from the cardia to the rectum; but it offers some varieties: thus the mucous membrane of the stomach is better provided with venous radicles, especially towards the pylorus, than that of the duodenum; and they always diminish in number towards the rectum, the quantity being much less in the larger than in the small intestines.

It follows therefore that the generally admitted opinion of anatomists that the arteries and veins communicate by means of an intermediate capillary system is not correct as regards the intestines; that intestinal absorption is sufficiently explained by the capillary attraction of the venous tubes; that this anatomical disposition accounts for the rapidity with which certain substances penetrate into the circulatory system, and which could not take place through the long circle of chyliferous lymphatics; and lastly, it also explains the occurrence of hæmatemesis and melæna, which are nothing more than venous hemorrhages of the gastro-intestinal mucous membrane.

*Revue Médicale. Juin*, 1841. (From *Memoriale della Med. Contemp.*)

---

*On the Presence of several Cysticerci in a Tumour having the appearance of a Boil.*
By Dr. Fournier, of Craon.

A child, six years old, had a tumour of the size of a hen's egg on the superior and lateral part of the neck, which had only appeared four days. It was red, hot, painful, of a conical form, and circumscribed. On examining it with care there appeared a small hole towards the base, in the middle of which a small white point was prominent, which had an almost imperceptible motion. A very limpid aqueous fluid flowed on pressure, and a particular but feeble sensation of *fremissement* was perceived. A species of clash (collision,) was distinguished by the ear, and the tumour, although red, hot, and painful, was soft and fluctuating, so that the presence of hydatids was diagnosticated. One of these worms was pressed out, and seven or eight more were removed by a small incision. They were afterwards recognized as cysticerci having a very small roundish head, supported by a contracted neck. The body was formed of imbricated rings perfectly visible to the naked eye; it was terminated by a small swelling, a kind of vesicle containing matter apparently albuminous. All performed some undulatory motions. The cure was complete on the seventh day.

*Journal des Connaissances Médico-Chirurgicales. Mai,* 1841.

*On the Temperature of Marine Invertebrata.* By VALENTIN and WILL.

THE authors have made observations on the proper temperature of seventeen kinds of marine invertebrata, comprising Polyps, Medusæ, Echinodermata, Helices, Cephalopods, and Crustacea. They found that all of them had a peculiar temperature varying with, but always somewhat surpassing, that of the medium in which they lived. The greatest difference amounted to 1°, the least to 0·1°; the former was observed in Pelagia denticulata, the latter in Aplysia leporina. With regard to the several classes the differences were among

| | |
|---|---|
| Polyps, on an average | +0·205 |
| Medusæ | +0·27 |
| Echinodermata | +0·40 |
| Helices | +0·46 |
| Cephalopods | +0·57 |
| Crustacea | +0·60 |

Which numbers prove an increase of proper temperature directly proportioned to the ascent in position in theanimal kingdom.

*Muller's Archiv.* 1840. Jahresbericht, S. xcviii.

---

*Experiments on the Development of Tubercles, and on the Action of Ferruginous Bread in preventing them.* By M. COSTER.

SIX rabbits of the same age (three weeks) and condition, were brought up from the 1st of April, 1838, in the manner described in the following numbers, 1, 2, 3, &c.

No. 1. Nourished in the open air, with a variety of herbs, as in its natural state, during one month.

No. 2. Nourished in a humid and dark cellar, the temperature varying only from 0 to 4°. All external motion impossible, the animal being inclosed in a very confined cage. Food: potatoes, turnips, and clover during five consecutive months.

No. 3. Placed in the same conditions with No. 2, but nourished alternately with the same vegetables, and with wheaten bread, with which carbonate of iron had been incorporated in the proportion of half a drachm to a pound of bread. A quarter of a pound of this bread was given in the day.

No. 4. Same external conditions as No. 1, but nourished as No. 3.

No. 5. Same conditions as No. 3, but instead of iron in the bread, iodide of potassium was employed in the proportion of 144 grains to the pound.

No. 6. Same as No. 2.

All were killed towards the end of August, when No. 1 was found healthy throughout. The tissues of the Nos. 2 and 6 were extremely flaccid, many glands engorged; tubercles existed to the number of three in the liver of one, and of two in the other; one in the spleen and many in the lung. Nos. 3 and 4 were healthy and well developed. No. 5 very lean, but without tubercles.

M. Coster states that he has experimented upon dogs, rabbits, guinea-pigs, and fowls, which he has submitted to the most injurious hygienic influences, and has separately combated these influences with iron, baryta, iodine, bromine, mercury, and tannin, and that hitherto the ferruginous bread has always prevented the production of tubercles.

*Bulletin de l'Académie Royale de Médecine. Avril* 15 *et* 30, 1841.

---

*On Spontaneous Combustion of the Human Body.* By Dr. JACOBS, of Eupen.

FROM twenty-eight cases of spontaneous combustion collected and analyzed by the author, he concludes:

1. That spontaneous combustion always occurs in living human beings, never after death nor in the lower animals. 2. The subjects were generally very old, the two youngest being fifty and twenty-nine years of age. 3. Women are most frequently the subjects, it having only occurred in *two* men. 4. It was once preceded by jaundice, once by a malignant ulcer on the head. 5. All the persons were alone at the time of the occurrence. 6. They led an idle life. 7. All were very fat, except three very lean females. 8. Almost all were intemperate. 9. Most frequently a light or some ignited substance was in the neighbourhood at the time of the accident. 10. The combustion proceeds very rapidly, and finishes in seven, three, and two hours, and even in one hour. 11. The flame, difficult to be extinguished by water, was very mobile, only destroying the objects placed very near, or in immediate contact with the burning body. 12. The room in which the combustion took place was usually filled with a thick vapour, and the walls covered by a black carbonaceous substance; the floor, ashes, and bones imbued with fat and fetid moisture. 13. The trunk was most frequently completely destroyed, some parts of the head and extremities usually remaining. 14. This combustion has occurred with only two exceptions during a cold temperature, in winter, and in the northern regions.

*Bulletin Général de Thérapeutique. Mai* 15 *et* 30, 1841.

---

*Injections of the Iris.* By Professor Grimelli, of Modena.

The author has made some experiments to support the opinions of Dr. Fario upon the vascular erectile structure of the iris. The substances which answer best for very fine injections of this organ are olive or walnut oil variously coloured, which penetrates into the most delicate vascular ramusculi without transuding through their coats, and preserves for a long time the parts which they impregnate. In injecting the bodies of children, Dr. Grimelli observed, that from being soft and much dilated, the iris became turgid, and contracted more than half its diameter, in the same manner as when the retina is affected by light during life. This fact appears to prove that the iris is composed of a union of vessels forming a disc, in the centre of which is the pupillary aperture, and the circumference of which is attached to the ciliary ligament. By the aid of the lens and microscope, we see that the very fine vessels which constitute the iris are disposed between the pupillary and ciliary circles, under the form of rectilinear and curvilinear radii, curved upon themselves and zigzag; agglomerated and united in an inextricable manner. We observe also some ramifications disposed in circles between the pupillary and ciliary circles, more or less near each other, and always few in number. It results from this disposition of the minute vessels, fixed towards the larger circle and moveable towards the lesser, that the sanguineous afflux and turgescence unfold the iris and contract the pupil, and that the return of blood and diminution of turgescence, fold again or wrinkle the membrane and dilate the pupillary aperture. Thus, contrary to the generally admitted opinion on the muscularity of the iris, as it appears to the author, this membrane is composed of a turgescible or erectile vascular tissue, in which arterial vessels predominate, and Dr. Grimelli is led by analogy to conclude that the muscles of the small bones of the ear are constituted in the same manner.

*Revue Médicale. Juin,* 1841. (From *Memoriale della Med. Contemp.*)

---

*On the Oxidation of Gelatine.* By M. Persoz.

M. Persoz has determined that when gelatine is submitted to an oxidizing agent it is susceptible of being transformed into hydrocyanic acid, ammonia, and carbonic acid, and a small quantity of one of the fat volatile and odoriferous acids, the existence of which was established by M. Chevreul. M. Persoz remarks that this fact leads to analytical researches to discover whether among the products of normal or abnormal cutaneous secretions, we cannot discover ammonia, hydrocyanic acid, or some one of its derivatives, as those composed of cyanogen and formic acid. He thinks that in certain cases of suppuration hydrocyanic acid may be formed, and states that M. Nonat has seen bandages and charpie tinted greenish-blue after having been for a long time in contact with the purulent matter of an abscess. This colouring may be attributed to Prussian-blue, but to verify and explain the fact, it will be necessary to observe the effect the pus of different wounds produces on pieces of linen impregnated with a salt of iron. *Gazette Médicale de Paris. Juillet* 24, 1841.

*On the Motions and Sounds of the Heart.* By Professor CRUVEILHIER.

A CHILD which had been born nine hours was brought under the notice of M. Cruveilhier The heart was outside the chest, having escaped through a perforation in the superior part of the sternum; it was thus as completely laid bare as though the sternum had been removed and the pericardium incised. Its surface was dry and so pale that M. Cruveilhier thought at first that it was still covered by its fibrous envelope. This opportunity for studying the motions and sounds of the heart was not neglected, and the following are the results.

A. *Motions of the Heart.*

1. Both ventricles contract simultaneously. Both auricles do the same.

2. The contraction of the ventricles coincides with the dilatation of the auricles, and the projection of blood into the arteries. The dilatation of the ventricles coincides with the contraction of the auricles, and the collapse of the arteries.

3. There are but two periods in the motions of the heart: the period of contraction and the period of dilatation; the period of repose, admitted by authors, is completely wanting. (Qy.—Would this be the same in a new-born as in an adult animal?—*Trans.*) Contraction immediately succeeds dilatation, and dilatation contraction.

4. It appears that the contraction and dilatation of the ventricles and auricles result from two opposed forces always active, which prevail alternately in a necessary and invariable order, like the alternate movements of a pendulum, or of a balance-wheel in perfect equilibrium.

5. The duration of the contraction of the ventricles is double that of their dilatation. If we divide into three equal periods the whole duration of the ventricular systole and diastole, we have two for contraction and one for dilatation. The same holds good with regard to the auricles.

6. During the contraction of the ventricles their surface becomes rugous, strongly folded, and as if shrivelled. The superficial veins swell; the fleshy columns of the right ventricle are delineated; the curved fibres of the summit (sommet) of the left ventricle, which alone constitutes the apex (pointe) of the heart, become more manifest.

7. During their contraction the ventricles contract in every diameter; the phenomena of shortening is the most sensible, but this is owing to the vertical diameter being the greatest. The summit of the left ventricle, or in other words the summit of the heart, describes a spiral movement from right to left, and from behind forwards.

8. It is to this slow, gradual, as it were successive, spiral contraction that the forward movement of the summit of the heart is owing, and the consequent percussion of the summit against the thoracic parietes. The ventricular systole is not accompanied by a projection of the heart forwards, it is the spiral contraction which exclusively determines the approach of the summit of the heart and the thoracic parietes.

9. The dilatation or diastole of the heart occurs in an abrupt instantaneous manner; it is so rapid and energetic that it appears to be an active motion of the heart. One cannot form an idea of the force with which the dilatation triumphs over pressure exercised upon this organ. The hand closed upon it is opened with violence.

10. The ventricular diastole is accompanied by a projection of the heart downwards. This motion was at its maximum when the child was placed vertically, and was very strongly marked.

11. The dilatation of the auricles is as abrupt as that of the ventricles; but its duration is marked by the duration of the ventricular systole. The contraction of the auricles is as brief as the systole of the ventricles.

12. During its dilatation the right auricle appears ready to burst, so much is it distended and so thin are its walls. The left auricle, smaller, longer, and thicker, does not exhibit the same phenomena, at least in so evident a manner.

B. *The Sounds of the Heart studied with regard to the Motions of the Heart, and the cause which produces them.*

1. The ear applied to the heart, either bare or covered by a piece of fine linen, recognized the double sound of the heart, of which the first was much more feeble than when it is heard through the thoracic parietes. It is evident therefore, on the one hand, that the cause of the double sound is inherent in the heart itself; and on the other hand, that the first sound is increased by the thoracic parietes. The feebleness of the first sound cannot be accounted for by the feebleness of the strokes of the heart, for they were very vigorous, and the infant extremely lively.

2. This double sound increased in proportion as the ear was carried from the apex of the heart towards the base, and *vice versa.* Thus it is at the base of the heart that we must seek for the cause of these sounds.

3. The finger applied to the origin of the pulmonary artery, (which is situated before the aorta, and completely conceals the latter,) experienced a perfectly distinct vibratory *fremissement,* which corresponded with the collapse of the artery and the dilatation of the ventricle. It was but feebly perceived at the moment of the dilatation of the artery and contraction of the ventricle.

4. As it was impossible to apply the ear directly to the perforation of the sternum, the finger was used as a stethoscope, and on applying the ear to any part of this finger, a sound of clapping (*bruit de claquement*) was heard very clearly. The same result was obtained on applying the ear to the angle formed by the junction of the second metarcarpal bone, with the first phalanx of this (the index) finger.

5. The double sound was sought in vain ; there was only one sharp sound, as brief as the second period. This sound coincided with the collapse of the artery, and consequently with the fall of the sigmoid valves, refolded by the column of blood. The use of the finger as a stethoscope was advantageous in combining the notions given by the sense of touch, with those by the ear.

6. The cause of the second sound therefore was very evidently in the vibratory thrill of the sigmoid, pulmonary, and aortic valves, thrown back by the column of blood.

7. In order to determine the cause of the first sound, and impressed with the idea that this sound had its source in the movements of the auriculo-ventricular valves, the finger was carried over the whole circumference of the base of the ventricles, in order to determine if there did not exist at the situation of the mitral and tricuspid valves some vibratory thrills analogous to those at the situation of the sigmoid valves. But none could be perceived, nor could any sound be heard by using the finger as a stethoscope over every accessible point of the base of the heart. Thus we were convinced that the mitral and tricuspid valves afforded no sound.

8. But if the first sound has not its seat in the auriculo-ventricular valves, if all the other parts of the heart are by themselves equally devoid of sound, and if the sounds which they communicate to the ear of the observer are sounds of transmission, is it not possible that the first sound has the same cause with the second, and results from the projection of the sigmoid valves by the column of blood, as the second does from their retrograde movement ? This idea appears to be converted into a demonstration by the following conclusions:

A. In this child the maximum intensity of the first sound was in the same situation with the maximum intensity of the second.

B. The first was exactly of the same nature as the second, except as to the intensity which was less, and the duration which was greater.

C. If the seat of the two sounds be the same, they would both be altered in all diseases of the sigmoid valves, which M. Cruveilhier says is the case in all the cases he has seen.

D. We should not be astonished that no sound accompanies the action of the auriculo-ventricular valves, for they are not free, but retained by the *chordæ tendineæ.* The ventricular contraction takes place in a successive manner, the apex of the heart moving towards the base; the elevation of these valves is consequently made in the same manner, and therefore without vibration. It is evident that when they are thickened, they will become more or less vibratile, and the resulting sound be confounded with that of the sigmoid valves.

E. It may be objected: if the sigmoid valves are the seat of the first sound, how is it that the greatest intensity is at the apex of the heart, and not at the base, the situation of these valves? The maximum of the first sound is at the apex of the heart when the ventricles contract with vigour, so that this point is strongly struck against the costal cartilages; but when the ventricles contract feebly, and consequently the percussion against the costal cartilages is feeble, M. Cruveilhier is convinced that it is behind the sternum, at the situation of the sigmoid valves, that the first sound is most intense. It is behind the sternum only that he has been able to recognize it in cases of dropsy of the pericardium.

The first sound is composed of two distinct phenomena: 1, the valvular sound; 2, the impulse of the apex of the heart against the costal walls. It is thus that in anæmia, chlorosis, and some organic diseases of the heart, the first sound is sonorous to such a degree as to have something of a metallic character, sometimes masking the second sound.

If the diaphragm were cartilaginous or osseous, resonant on percussion, it would be over the xiphoid cartilage, at the moment of the ventricular diastole, that the second sound would be most intense, in consequence of the abrupt projection downwards of the heart during the diastole.

[Without offering any opinion as to the validity of M. Cruveilhier's conclusion respecting the cause of the sounds of the heart, we may observe that it obviates one difficulty which we have always felt in assenting to all the other theories which attributed the two sounds to the action of different parts. This objection we stated many years ago in the following words: "In reference to this opinion of M. Bouillaud [that both sounds are owing to the play of the valves of the heart], as also to that of Dr. Hope [an early opinion, that the first sound was caused by the ventricular systole and the second by the ventricular diastole], I would observe that they both possess a degree of probability in my mind over all those which attribute the two sounds to *two* different causes. Although certainly characteristically different, yet the two sounds have so great a similarity, and are so allied in time and place, that I cannot readily bring my mind to believe that they do not both depend upon one and the same cause slightly modified, or, at least, on the different play of the same parts."—*Note to the Fourth Edition of the Translation of Laennec.*—ED.]

*Gazette Médicale de Paris.* 7 *Aout*, 1841.

